# Exposure to atheroma-relevant 7-oxysterols causes proteomic alterations in cell death, cellular longevity, and lipid metabolism in THP-1 macrophages

**DOI:** 10.1371/journal.pone.0174475

**Published:** 2017-03-28

**Authors:** Liam J. Ward, Stefan A. Ljunggren, Helen Karlsson, Wei Li, Xi-Ming Yuan

**Affiliations:** 1 Occupational and Environmental Medicine Center, and Department of Clinical and Experimental Medicine, Linköping University, Linköping, Sweden; 2 Division of Obstetrics and Gynaecology, and Department of Clinical and Experimental Medicine, Linköping University, Linköping, Sweden; Università degli Studi di Milano, ITALY

## Abstract

The 7-oxysterols are recognised as strong enhancers of inflammatory processes in foamy macrophages. Atheroma-relevant 7-oxysterol mixtures induce a mixed type of cell death in macrophages, and trigger cellular oxidative stress responses, which mimic oxidative exposures observed in atherosclerotic lesions. However, the macrophage proteome has not previously been determined in the 7-oxysterol treated cell model. The aim of the present study was to determine the specific effects of an atheroma-relevant 7-oxysterol mixture on human macrophage proteome. Human THP-1 macrophages were exposed to an atheroma-relevant mixture of 7β-hydroxycholesterol and 7-ketocholesterol. Two-dimensional gel electrophoresis and mass spectrometry techniques were used to analyse the alterations in macrophage proteome, which resulted in the identification of 19 proteins with significant differential expression upon oxysterol loading; 8 increased and 11 decreased. The expression patterns of 11 out of 19 identified significant proteins were further confirmed by tandem-mass spectrometry, including further validation of increased histone deacetylase 2 and macrophage scavenger receptor types I and II expressions by western blot analysis. Identified proteins with differential expression in the cell model have been associated with *i*) signalling imbalance in cell death and cellular longevity; *ii*) lipid uptake and metabolism in foam cells; and *iii*) inflammatory proteins. The presented findings highlight a new proteomic platform for further studies into the functional roles of macrophages in atherosclerosis, and present a cell model for future studies to modulate the macrophage proteome by potential anti-atherosclerotic agents.

## Introduction

Oxysterols, oxidative derivatives of cholesterol, are recognised as strong enhancers of inflammatory processes and have increased biochemical reactivity when compared to cholesterol [1]. These properties have made oxysterols a point of interest in pathological diseases associated with inflammation, such as atherosclerosis. Endogenous oxysterols are of key relevance, where non-enzymatic production is the major source of endogenous oxysterols, such as 7-ketocholesterol and 7β-hydroxycholesterol [2].

In atherosclerotic lesions oxysterols are abundantly present, as mixtures, and have shown cytotoxicity in a number of cell type within the vessel wall [3, 4]. In addition, oxysterols have displayed specific effects on cell types associated with the onset of atherosclerosis, contributing to endothelial cell dysfunction and the progression of atherosclerosis, by stimulating the formation and subsequent apoptosis of macrophage foam cells. In our previous study [5], it was demonstrated that oxysterol mixtures induce a mixed type of cell death, with the presence of both apoptotic and necrotic cells, an overproduction of reactive oxygen species, and a decrease of reduced thiols (including glutathione) [5, 6]. These adverse effects are hallmarks of cellular alterations which contribute to the development and instability of atherosclerotic lesions, highlighting the physiological relevance of oxysterol mixtures in inducing these effects. Moreover, a synergistic cytotoxic effect was revealed between 7-ketocholesterol and 7β-hydroxycholesterol, which are among the most abundant oxysterols found in oxidised-LDL (oxLDL) and atherosclerotic lesions [6].

Macrophages are crucial in atherogenesis by promoting the inflammatory response and the formation of foam cells. Foam cell formation can occur with the continued uptake of oxLDL that is abundant in oxysterols. Atheroma-relevant mixed oxysterols have induced up-regulations of monocyte chemotactic protein-1, CD36 and matrix metalloproteinase-9 in macrophages that can contribute to inflammation, lipid uptake and plaque vulnerability, respectively, during atherosclerosis [7–9]. The human THP-1 monocyte cell line, and differentiated macrophages, is a well-established model for the mimicry of primary vascular monocytes/macrophages in terms of physiology, immunology and atherosclerosis [10]. In addition, THP-1 macrophages have been successfully polarised to M1 or M2 macrophages and seen to display remarkably similar expression patterns to those polarised from isolated primary monocytes [11]. In addition, the THP-1 cell line has been used to test potential anti-atherosclerotic agents, whereby the pro-apoptotic effects of 7-ketocholesterol were shown to decrease upon treatment with a dietary phytochemical that maintains cell redox balance and reduces oxidative stress [12].

Proteomics, specifically gel-based, has proved to be a successful methodology to determine the protein alterations in macrophages when exposed to oxLDL [13–15]. OxLDL-loading in macrophages has resulted in observed alterations in apoptosis-related proteins and cellular stress markers [14, 15]. However, proteomics has not previously been used to determine the specific effects of an atheroma-relevant oxysterol mixture on the human macrophage proteome.

The aim of this study is to identify and characterise the effect of an atheroma-relevant oxysterol mixture on the proteome of THP-1 macrophages in the established cell stress/death model, which mimics oxidative exposures observed in atherosclerotic lesions [6]. The hypothesis is that the macrophage proteome alters significantly upon exposure to the atheroma-relevant oxysterol mixture, particularly in the expression of proteins related to cell death, cellular longevity and inflammation. If so, the cell model can be used as an insightful proteomic platform to investigate pathophysiological effect of oxysterols in atherosclerosis. Here we show that oxysterol treatment of THP-1 derived macrophages elicit a number of differential protein expressions, where three groups of proteins can be highlighted due to their functional associations with: *i*) signalling imbalance in cell death and cellular longevity; *ii*) lipid uptake and metabolism in foam cells; and *iii*) inflammatory marker proteins.

## Materials and methods

### Cell culture and treatment

Human THP-1 monocytes (ATCC, VA, USA) were maintained in RPMI-1640 medium with glutamate supplemented with 10% FBS, 100 U/mL penicillin and 100 μg/mL streptomycin (GIBCO, UK). Cells were cultured in 5% CO_2_ humidified atmosphere at 37°C and divided twice a week. For all experiments cells were seeded at an approximate density of 5 x 10^5^/mL. Macrophage differentiation was induced by incubation with 300 nM phorbol myristate acetate (PMA; Sigma-Aldrich, MO, USA) for 24 h. Culture medium was then replaced and cells cultured for an additional 48 h. Experiments were performed with a mixed treatment of 7β-hydroxycholesterol and 7-ketocholesterol (Sigma-Aldrich) at a ratio of 1:1.8 (28 μM; referred to as 2mix) for 24 hr, previously tested within our group to induce an apoptotic state [5, 16]. Control experiments were performed using untreated, cholesterol, ethanol, and 7-ketocholestrol individually. The concentrations of oxysterols used in the experiments are substantially lower than those reported to exist in human atherosclerotic plaque [3, 17], although greater than normal healthy physiological conditions, and provide a viable cell model to analyse macrophage dysfunction during atherosclerosis [4].

### Annexin V/DAPI staining

Apoptotic cells were assayed by detection of phosphatidylserine exposure using fluorescence microscopy following Annexin V (AV) (Roche Mannheim, Germany) staining. Briefly, control and treated cells were washed once with PBS, and stained for 10 minutes on ice with AV and nuclear counterstained with DAPI.

### Protein extraction

Protein extraction was performed as previously described [18], briefly; macrophages were washed in PBS thrice, to remove bovine protein contaminants, then collected and homogenised in protease inhibitor cocktail solution (CompleteMini; Roche, Switzerland). Supernatant was collected and protein precipitation, using 10% trichloroacetic acid in acetone, was performed at -20°C for 2 h. Samples were centrifuged at 4°C, 19800g for 10 min and supernatant discarded, remaining pellet was washed in 20 mM dithiothreitol in acetone and centrifuged as before and supernatant discarded. Samples were then reconstituted in 250 μL of urea sample solution, containing; 9 M urea, 1% dithiothreitol, 4% CHAPS, 2% IPG buffer pH 3–10 (GE Healthcare, UK) and 1% bromophenol blue. Aliquots were taken for protein concentration determined by 2-D Quant Kit (GE healthcare) performed to manufacturer guidelines.

### Two-dimensional gel electrophoresis (2-DE)

2-DE was performed horizontally using an IPGphor and Multiphor (GE Healthcare) setup according to a method previously described by Görg, *et al*. [19], used by our group in the analysis of macrophage cell samples [18]. Macrophage protein samples, approximately 150 μg of protein per sample, were separated by 2-DE, with the following experimental replicates: 5 untreated control; 5 2mix treated; 3 7-ketocholesterol treated; 3 cholesterol treated; and 3 ethanol treated. First dimension isoelectric focussing was performed on pH 3–10 non-linear IPG strips (GE Healthcare) for 46000 Vh. Second dimension was performed using home cast homogenous gels (SDS-PAGE; T = 14%, C = 1.5%) and run overnight. 2-DE gels were stained using silver nitrate, according to the protocol set by Shevchenko, *et al*. [20]. Stained gels were imaged using a charge-coupled device camera and VersaDoc 4000MP system (Bio-Rad Laboratories, CA, USA) and gels spots matched and quantified, as parts per million (ppm) of total gel density, between gels using PD-Quest v8.0.1 (Bio-Rad Laboratories). Statistical analysis of matched protein spots between control untreated cells and treatments; 2mix, 7-ketocholesterol, cholesterol and ethanol was performed using a non-parametric Mann-Whitney *U* test (SPSS v21; IBM, UK), where significance was set at p ≤ 0.05.

### MALDI-TOF MS

Protein spots, which were deemed to have significant differential expression, were excised from the 2-DE gels and silver nitrate staining removed according to a previously described method [18]. In-gel tryptic digestion was performed using porcine trypsin solution (5 μg/mL in 25 mM ammonium bicarbonate; Promega, WI, USA) with overnight incubation at 37°C. Peptide containing supernatants were transferred and dried via vacuum centrifugation (SpeedVac; Savant, NY, USA), and then dried peptides reconstituted in 5 μL of 0.1% trifluoroacetic acid. Reconstituted peptides were mixed with 0.19 M 2,5-dihydroxybenzoic acid (Sigma-Aldrich) in 70% acetonitrile/0.3% trifluoroacetic acid, as matrix, at a ratio of 1:1, and spotted on a stainless-steel target plate. A standardised protein mix (Calibration Mixture 1 and 2; AB SCIEX, MA, USA) was spotted as external calibration. Peptide mass-fingerprinting was performed using MALDI-TOF MS (Voyager DE PRO; Applied Biosystems, CA, USA).

Database searching was performed on the major peaks in the spectra, generated using Voyager Data Explorer software (v5.1; Applied Biosystems), with post-internal calibration using known trypsin autolysis peaks (*m/z* 842.5100 and 2211.1046). Major peak lists were submitted to the MS-Fit search engine (https://prospector.ucsf.edu) and searched within the Swiss-Prot and UniProt databases. Search parameters were set as; *Homo sapiens*, mass tolerance < 75 ppm, maximum one missed cleavage, constant modification of carbomidomethyl on cysteine and variable modification of oxidation on methionine.

### Tandem-mass spectrometry (MS/MS)

Protein identities were also confirmed by MS/MS (LTQ Orbitrap Velos Pro, Thermo Fisher Scientific, MA, USA). THP-1 samples, untreated control and 2mix treated each in triplicates, were lysed in 6M urea / 2M thiourea in 25 mM ammonium bicarbonate followed by sonication. THP-1 samples were reduced and alkylated, via dithiothreitol (0.25 M) and iodoacetamide (0.75 M) treatment, before running through a 3 kDa cut-off filter (Amicon Ultra 3K device; Merck-Millipore, Germany) to concentrate and remove urea. Protein digestion was performed with trypsin (1:25 trypsin/protein) and resulting peptide samples dried before reconstitution in 0.1% formic acid in water. Samples were loaded at a total protein concentration of 250 ng and separated using nanoflow HPLC system (Easy-nLC; Thermo Fisher Scientific) with a C18 column (100 mm x 0.75 μm; Agilent Technologies, CA, USA) and loaded into the MS with a 1.5 h gradient. Spectra were processed using MaxQuant (v1.5.3; Max Planck Institute of Biochemistry, Germany) to search against the human protein database (UniProt, downloaded 17^th^ January 2015) using 6 ppm mass tolerance for MS and 0.5 Da for MS/MS. Modifications included constant modification of carbomidomethyl on cysteine and variable modification of oxidation on methionine. Peptides with a false-discovery rate of less than 1% were retained and proteins with at least 2 unique peptide were considered identified. Label-free quantification, using an in-software algorithm, was used to represent the amount of protein abundance within samples.

### Western blot analysis

Control and 2mix treated THP-1 whole cell protein extracts (25 μg per sample) were separated by SDS-PAGE (homogenous gels, T: 15% and C: 1.5%) using the mini-protean II electrophoresis cell (Bio-Rad Laboratories). Separated proteins were blotted onto Immuno-Blot PVDF Membrane (Bio-Rad Laboratories) and membranes blocked with Tris-buffered saline (TBS) with 5% non-fat dried milk. Membranes were then incubated overnight in TBS-Tween 20 (TBS-T), with 2% non-fat dried milk, together with primary antibodies for anti-HDAC2 (1μg/mL; Nordic Biosite, Sweden), or anti-MSR1 (2μg/mL; Merck-Millipore, Germany). Membranes were washed with TBS-T followed by incubation with HRP-conjugated secondary anti-rabbit antibody for 1 hour. Antigen/antibody conjugates were illuminated using enhanced chemiluminescence (ECL) solution (GE Healthcare) and visualised using charge-coupled device camera. Resulting blots were quantified using ImageJ software (National Institute of Health, USA).

## Results

### Exposure to 2mix induces apoptosis and autophagy in THP-1 differentiated macrophages

**Fig 1:**
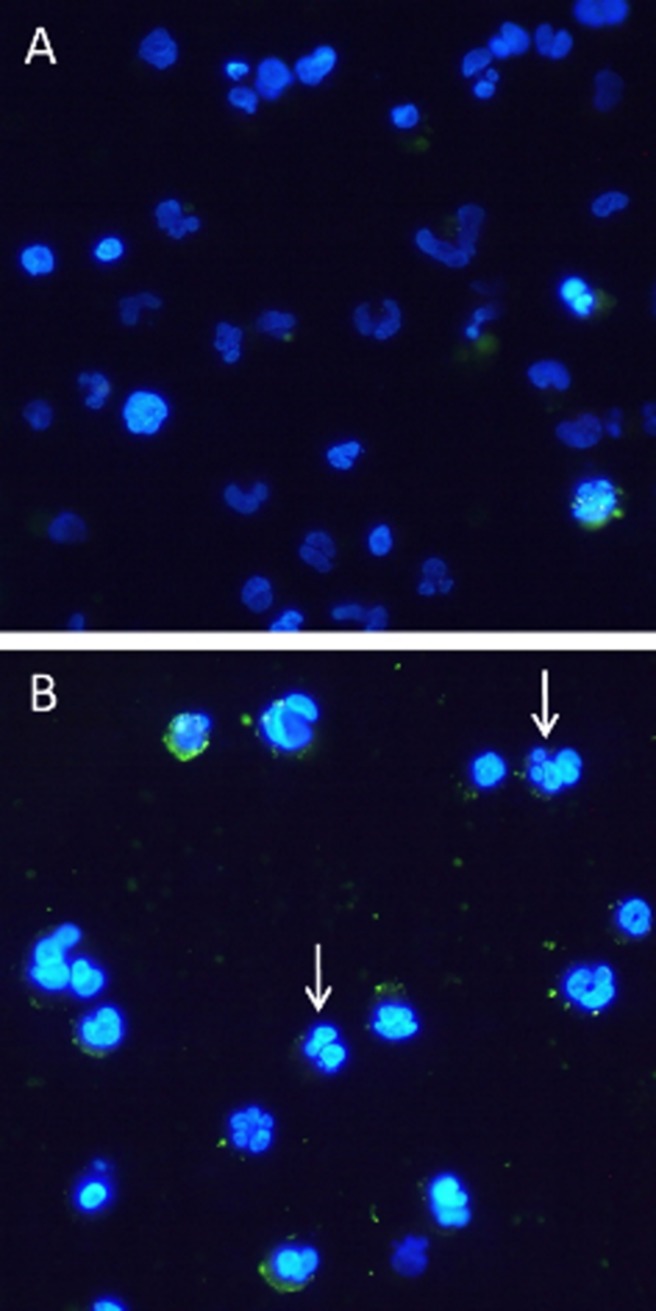
Exposure to 2mix induces apoptosis in THP-1 differentiated macrophages. Cells were treated for 24 h with PBS (A) or 2mix (B; a mixed treatment of 7-ketocholesterol and 7β-hydroxycholesterol), and then stained with Annexin V + DAPI for apoptosis. White arrows in (B) indicate nuclear fragmentation (magnification x63).

THP-1 differentiated macrophages were cultured for 24 h with or without 2mix treatment, a mixed treatment of 7-ketocholesterol and 7β-hydroxycholesterol. To confirm whether these cells were in an apoptotic state, cells were stained with AV (membrane green fluorescence) and nuclear stained with DAPI (blue fluorescence). As compared to control cells (Fig 1A), 2mix treated cells (Fig 1B) showed cell membrane positive staining of AV, nuclear shrinkage and condensation as well as nuclear fragmentation (Fig 1B, arrows). These are consistent with previous studies [16, 21]. It is known that 7-ketocholesterol and 7β-hydroxycholesterol at 20 μg/ml induces remarkable increases in cellular concentrations of 7-ketocholesterol and 7β-hydroxycholesterol (781 to 3468 times higher than in untreated cells), and apoptosis which are not correlated with oxysterol accumulation in the lipid raft [22]. We recently showed that exposure to 7-oxysterols in the cell model induced autophagic vacuole synthesis in the form of increased autophagy marker microtubule-associated protein 1A/1B-light chain 3 (LC3) and LC3-phosphatidylethanolamine conjugate (LC3-II). This led to an accumulation of p62, indicating a reduction in autophagic vacuole degradation [23].

### Exposure to 2mix induces significant alterations in the THP-1 macrophage proteome, specifically within proteins related to cell death and cellular longevity, lipid metabolism, and inflammation

THP-1 macrophage protein samples were separated using 2-DE and stained using silver nitrate, all gels with the exception of one 2mix treated gel stained successfully. Over 400 proteins spots per gel were detected and matched between gels. Statistical analysis was performed, initially between untreated control and 2mix treated samples, using relative abundance per matched protein spot using a non-parametric Mann Whitney *U*-test, whereby 39 protein spots were detected to display significant (p ≤ 0.05) differential expression between the untreated control and 2mix treated groups; 16 protein spots were more abundant and 23 less abundant with 2mix treatment. Significant proteins spots were selected for identification by MALDI-TOF MS analysis where 20 proteins spots, corresponding to 19 unique proteins, were successfully identified (S1 Table).

**Fig 2:**
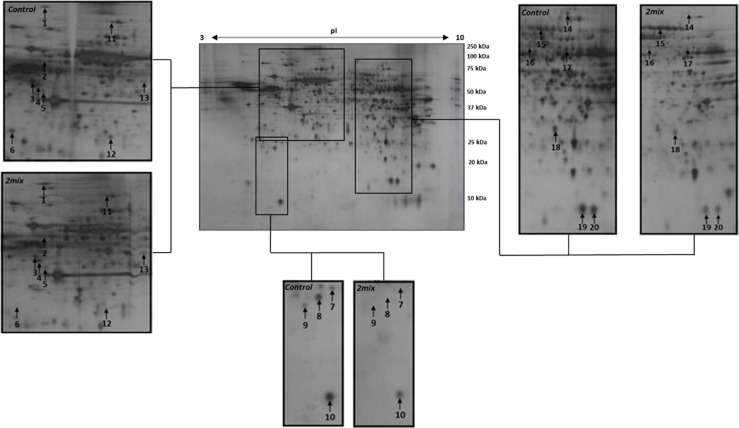
Exposure to 2mix induces significant alterations in macrophage proteome. Representative two-dimensional gel electrophoresis images show alterations in protein spot intensities between control and 2mix treated THP-1 macrophages. Central image is of an untreated control macrophage sample and outlying frames are representations of the differences between control and oxysterol (2mix) treatments. Protein spots with significant differential expression between treatments have been numbered, which correspond to identities presented in Table 1.

Fig 2 displays the identified gel spot positions in the 2-DE gels with representative gel image comparisons between the control and 2mix treated groups. Relative quantification of the identified gel spots resulted in 8 protein spots being more abundant, and 12 protein spots less abundant, upon treatment with 2mix (Table 1). Observed differences in representative images (Fig 2) may differ from reported fold changes (Table 1) due to the systematic normalisation of quantitative data with regards to extent of protein loading and silver nitrate staining.

**Table 1:** Oxysterol treated THP1 macrophages display 19 significantly altered proteins as identified by 2-DE and MALDI-TOF MS. Significantly altered protein spots, between control and oxysterol (2mix) treated THP-1 macrophage samples, were excised for identification. Fold change represents the extent of differed spot intensity from control to oxysterol treated THP-1 macrophage. Spot numbers correspond to those spots highlighted in Fig 2. Statistical analysis was performed, between matched protein spots, via non-parametric Mann-Whitney U test, and *p ≤ 0.05 were considered significant.

Spot n#	Protein name	UniProt accession n#	Gene name	Spot intensity (mean±SEM)		Fold change	p-value
				Control	2mix		
**1**	Hypoxia upregulated protein 1	Q9Y4L1	HYOU1	2303.60 (429.92)	5010.14 (1557.54)	+ 2.17	0.050
**2**	Zinc finger protein 202	O95125	ZNF202	1032.07 (178.14)	1493.59 (78.36)	+ 1.45	0.050
**3**	ATP synthase subunit β	P06576	ATP5B	5064.12 (1048.99)	10328.50 (2389.66)	+ 2.04	0.050
**4**	Protein disulphide-isomerase A6	B7Z254	PDIA6	8915.74 (1166,11)	4597.38 (612.98)	- 1.94	0.050
**5**	Macrophage scavenger receptor types I and II	P21757	MSR1	1011.93 (141.77)	2092.57 (259.68)	+ 2.07	0.014
**6**	Geranylgeranyl transferase type 2 subunit β	P53611	GGTB	3442.64 (375.26)	872.28 (337.40)	- 3.95	0.014
**7**	Rho GDP-dissociation inhibitor 2	P52566	GDIA2	2527.57 (390.22)	905.38 (540.56)	- 2.79	0.050
**8**	Glyoxalase 1	Q04760	GLO1	8538.05 (525.28)	2566.13 (1098.30)	- 3.33	0.014
**9**	Phosducin-like protein	Q13371	PDCL	2997.81 (491.02)	695.70 (337.11)	- 4.31	0.014
**10**	Galectin-1	P09382	LGALS1	15824.88 (2569.12)	8390.51 (2916.02)	- 1.89	0.050
**11**	Alpha-glucosidase II subunit α	Q14697	GANAB	363.42 (35.53)	798.48 (107.59)	+ 2.20	0.014
**12**	Annexin A4	P09525	ANXA4	3704.58 (245.30)	2309.09 (404,64)	- 1.60	0.027
**13**	Tryptophan–tRNA ligase	P23381	WARS	506.60 (64.26)	1120.80 (384.27)	+ 2.21	0.014
**14**	Nuclear factor of activated T-cells, cytoplasmic 1	O95644	NFATC1	1284.03 (106.59)	683.89 (81.13)	- 1.88	0.014
**15**	Tyrosine-protein phosphatase non-receptor type 11	Q06124	PTPN11	3119.91 (565.87)	6836.54 (1566.17)	+ 2.19	0.027
**16**	Histone deacetylase 2	Q92769	HDAC2	878.90 (65.63)	2001.57 (441.73)	+ 2.28	0.050
**17**	Adenylyl cyclase-associated protein 1	Q01518	CAP1	5319.85 (368.04)	1909.90 (260.74)	- 1.63	0.050
**18**	Syntenin-1	O00560	SYCL	7861.13 (1496.26)	2827.89 (716,51)	- 2.78	0.014
**19**	Cyclophilin A	P62937	CYPA (#1)	15743.20 (4109.99)	3678.67 (1545.23)	- 4.28	0.027
**20**	Cyclophilin A	P62937	CYPA (#2)	14644.81 (3465.66)	5260.25 (1321.07)	- 2.78	0.050

**Fig 3:**
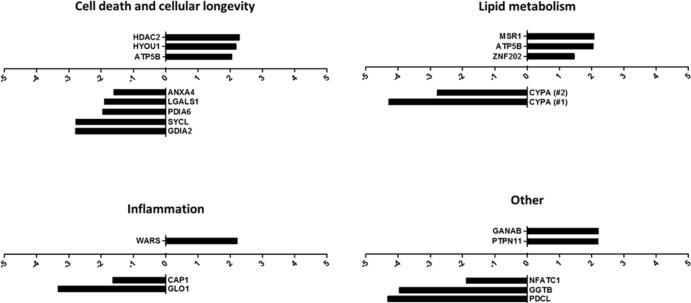
2mix exposure induces significant alterations in cell death and cellular longevity, lipid metabolism, and inflammation related proteins in THP-1 macrophages. Relative fold change of significantly altered protein spots, identified by means of two-dimensional gel electrophoresis quantification. Gene name annotations refer to protein identities presented in Table 1. Fold change was determined using the averaged relative expression (ppm of total gel density) from the two-dimensional gel electrophoresis quantification of 5 control THP-1 macrophage gels and 4 oxysterol (2mix) treated THP-1 macrophage gels. Proteins have been divided into distinct groups according to function: cell death and cellular longevity, lipid metabolism, inflammation and other.

Identified proteins were grouped by function into one or more of the following functional groups: cell death and cellular longevity, lipid metabolism, inflammation and other proteins (Fig 3). Proteins within these groups were ranked by fold change, control to 2mix treated, from the greatest positive fold change to the greatest negative fold change (Fig 3). The protein spot with the greatest positive fold change was histone deacetylase 2 (HDAC2 = +2.28), and the greatest negative fold change was phosducin-like protein (PDCL = -4.31). With the exception of zinc finger protein 202 (ZNF202 = +1.45), all proteins with a positive fold change were observed to have at least a doubled (≥ +2) level of expression in the 2mix treated THP-1 samples. Whereas, out of the 11 proteins that displayed a negative fold change only 6 proteins showed a fold change ≤ -2, with both cyclophilin A (CYPA) protein spots being lower (CYPA#1 = -4.28; CYPA#2 = -2.78) (Table 1 and Fig 3).

Confirmation that protein spot alterations were the effect of 2mix was performed by analysis of THP-1 samples treated separately to 7-ketocholesterol, cholesterol and ethanol. Statistical analyses were performed between untreated controls and each individual treatment (7-ketocholesterol, cholesterol or ethanol), and then between each treatment and 2mix treated samples. Results did not display any significant alterations in the identified protein spots which were not then significantly exacerbated further, in the same direction, by 2mix treatment (S2 Table**).** Interestingly, no protein spots showed significant alterations when comparing the untreated control and cholesterol treated group.

### 2mix-induced alterations of the THP-1 macrophage proteome are validated by MS/MS analysis and western blot analysis

**Fig 4:**
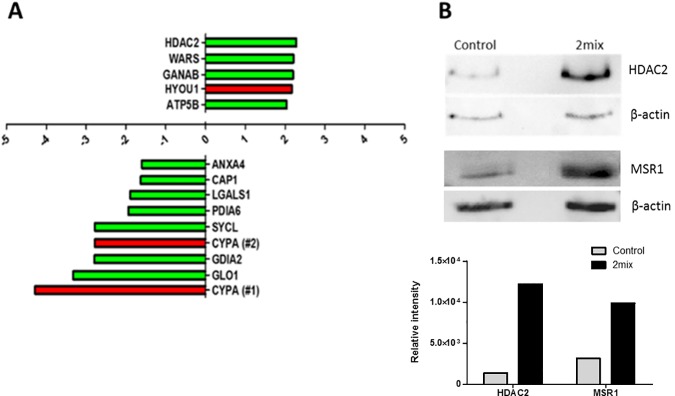
2mix induced alterations of cell death and cellular longevity, and inflammation related proteins in the THP-1 macrophage proteome are further validated by tandem-mass spectrometry analysis and western blot analysis. (A) Confirmation of 2mix-induced protein alterations in THP-1 macrophages by tandem-mass spectrometry. Fold change of significantly altered protein spots from oxysterol treated THP-1 macrophages identified by means of two-dimensional gel electrophoresis quantification and further validated by tandem-mass spectrometry analysis. Fold change was determined using the averaged relative expression (ppm of total gel density) from two-dimensional gel electrophoresis quantification of 5 control treated THP-1 macrophage gels and 4 oxysterol (2mix) treated THP-1 macrophage gels (Table 1). Validation of expression direction was performed by tandem-mass spectrometry analysis; where proteins highlighted in green confirm trends of expression, and those highlighted in red display opposing trends in expression between methods (Table 2). Gene name annotations refer to protein identities presented in Tables 1 and 2. (B) Western blot analysis of histone deacetylase 2 (HDAC2) and macrophage scavenger receptor types I and II (MSR1) expression in control and 2mix treated THP-1 cells. 2mix induces increased abundance of both HDAC2 and MSR1 in the THP-1 macrophage.

Further validation was performed using MS/MS analysis, which identified over 1200 proteins (data can be accessed via PRIDE database: PXD004304). Table 2 presents those 13 proteins which were identified using both MALDI-TOF MS and MS/MS methods, which includes all proteins from the cell death and cellular longevity, and inflammation functional groups. Quantification of MS/MS data between control and 2mix treated samples, when compared to 2-DE results, corroborates the direction of expression in 11 proteins, with the exceptions of hypoxia upregulated protein 1 (HYOU1) and CYPA (Table 2 and Fig 4A).

**Table 2:** Secondary analysis by tandem-mass spectrometry confirms the identification of 13 protein identities, and the expression pattern of 11 proteins in oxysterol treated THP-1 macrophages. Analysis of protein extracts from THP-1 macrophages, control and oxysterol (2mix) treated, by liquid chromatography-tandem mass spectrometry (nLC-MS/MS). Proteins identified by both nLC-MS/MS and 2-DE/MALDI-TOF MS are presented.

Protein name	UniProt accession n#	Gene name	Sequence coverage (%)	Normalised abundance (% (±SEM))^a^		Alteration
				Control	2mix^b^	
Adenyl cyclase-associated protein 1	Q01518	CAP1	39.8	0.194 (0.012)	0.169 (0.018)	-12.9%
Alpha-glucosidase II subunit α	Q14697	GANAB	47.6	0.316 (0.049)	0.376 (0.063)	+19.0%
Annexin A4	P09525	ANXA4	41.4	0.105 (0.003)	0.077 (0.039)	-26.7%
ATP synthase subunit β	P06576	ATP5B	73.7	1.741 (0.512)	2.126 (0.342)	+22.1%
Cyclophilin A	P62937	CYPA	71.5	0.705 (0.026)	0.712 (0.034)	+1.0%
Galectin-1	P09382	LGALS1	74.8	0.422 (0.104)	0.364 (0.074)	-13.7%
Glyoxalase 1	Q04760	GLO1	44.6	0.016 (0.008)	0.014 (0.007)	-12.5%
Histone deacetylase 2	Q92769	HDAC2	15.9	0.017 (0.008)	0.019 (0.009)	+11.7%
Hypoxia upregulated protein 1	Q9Y4L1	HYOU1	39.8	0.141 (0.034)	0.133 (0.022)	-5.7%
Protein disulphide-isomerase A6	B7Z254	PDIA6	53.1	0.279 (0.010)	0.272 (0.024)	-2.5%
Rho GDP-dissociation inhibitor 2	P52566	GDIA2	58.7	0.179 (0.005)	0.135 (0.023)	-24.6%
Syntenin-1	O00560	SYCL	22.5	0.012 (0.005)	0.009 (0.004)	-25.0%
Tryptophan–tRNA ligase	P23381	WARS	16.3	0.009 (0.005)	0.010 (0.005)	+11.1%

^*a*^ data normalised as a percentage of the total abundance of all identified proteins per sample, control or 2mix, and values averaged from triplicate analyses using nLC-MS/MS using label-free quantification.

^*b*^ oxysterol mixture of 7β-hydroxycholesterol and 7-ketocholesterol

Cell death and cellular longevity related protein alterations were validated, by MS/MS, for 2 proteins with increased abundances, HDAC2 and ATP synthase subunit β (ATP5B), and all proteins with decreased abundances. ATP5B and HDAC2 were found to increase in abundance by approximately 22.1% and 11.7%, respectively, between control and 2mix treated cells (Table 2). The most pronounced decreases in abundance by MS/MS analysis were found in annexin A4 (-26.7%), syntenin-1 (-25.0%) and Rho GDP-dissociation inhibitor 2 (GDIA2; -24.6%).

MS/MS analysis has validated the direction of expression for all proteins within the inflammation functional group. Increased abundance of tryptophan t-RNA ligase (+11.1%), and decrease abundance of both adenylyl cyclase-associated protein 1 (CAP1; -12.9%) and glyoxalase 1 (-12.5%) (Table 2).

In addition to the validation by MS/MS, western blot analysis was also performed for selected proteins HDAC2 and MSR1 (Fig 4B). Western blot results for HDAC2 are in agreement with both 2-DE and MS/MS results displaying an increased abundance on 2mix treatment. Also in agreement with 2-DE results the increased abundance of MSR1 on 2mix treatment was found by western blot (Fig 4B).

Comparing the two quantifications, 2-DE and MS/MS, it can be observed that the difference in expression between control and 2mix treated samples is smaller in the MS/MS quantification. This highlights the major advantage of 2-DE methods, when the protein is abundant and within the defined detectable mass range, where individual isoforms of proteins can be quantified. Whereas, with MS/MS techniques multiple isoforms are not distinguishable and quantified cumulatively. In addition, MS/MS results are highly dependent on the LC separation. MS/MS techniques are advantageous in that they are highly-sensitive in terms of protein identification, as they are not as limited to protein mass as 2-DE methods, which is clearly demonstrated in the number of proteins that can be identified by either method. This may well account for the opposing expression patterns observed between methods with HYOU1 and CYPA (Fig 4A), where significant alterations seen in the 2-DE pattern are likely isoform and/or modified protein specific.

## Discussion

Macrophage-derived foam cells are induced by the uptake of oxLDL that are abundant in 7-oxysterols. In order to understand molecular events driving this process it is of importance to identify and establish the proteomic profiles of 7-oxysterol-induced foamy macrophages. The present study for the first time investigates the effect of oxysterol loading, in an atheroma-relevant mixture, on the macrophage proteome and the functional implications. A total of 19 proteins displayed significant alterations in oxysterol-loaded macrophages using 2-DE methods, of which 13 proteins were further validated using MS/MS. Comparing the results from these two methods, the expression patterns of three functional groups of proteins were established in the oxysterol-treated macrophage model.

### Proteins related to cell death and cellular longevity

Oxysterol treatment of THP-1 monocytes/macrophages, is an established model for analysing cell death, apoptosis, and autophagy. In accordance with this, the first group of significantly altered proteins identified were associated with cell death and cellular longevity. Within this group three protein were more abundant: HDAC2, HYOU1, and ATP5B; and five less abundant: annexin A4, galectin-1, PDIA6, GDIA2, and syntenin-1 (Fig 3).

HDAC2 expression was found significantly increased upon oxysterol treatment in the 2DE analysis, which was also confirmed and validated by both MS/MS analysis and western blot analysis. Specific roles for histone deacetylases in atherogenesis have yet to be elucidated, however described roles in the control of cell cycle and apoptosis have been reported. Modified HDAC2, by post-translational sumoylation, has shown to specifically deacetylase p53 and although THP-1 cells are p53 mutated observed production of p53 has been reported upon treatment with oxysterols [16]. Disruption of p53-dependent gene expression of proteins controlling the cell cycle can attenuate DNA damage-induced apoptosis [24]. HDAC’s have also been studied with respect to autophagy, whereby the regulation of HDAC’s mediated the induction of autophagic gene expression and autophagosome formation [25]. These findings indicate that the increased abundance of HDAC2 in our model may restrict p53 function and attenuate DNA damage-induced apoptosis and are responsible for up-regulated autophagy in the cell model observed previously [26].

HYOU1 expression has seen to be induced upon treatment with oxysterols and oxLDL in cultured cells, with increased endoplasmic reticulum stress and apoptosis markers [27, 28]. In advanced atherosclerotic lesions immunostaining has localised the expression of HYOU1 to foam cells, and the plaque core, where it is believed to be involved in cellular survival mechanisms, reducing apoptosis and endoplasmic reticulum stress by blocking calcium signalling [27, 28]. The increase in HYOU1 expression in the 2-DE results may be indicative of an active self-defence mechanism against oxysterol-induced stress.

ATP5B has been associated with increased mitochondrial permeability under stressful conditions leading to cell death [29]. In oxysterol treated THP-1 macrophages ATP5B expression was increased, possibly indicating ATP synthase complex dissociation, contributing to the apoptotic macrophages observed.

Oxysterol loading in THP-1 macrophages resulted in the significant decrease in abundance of several proteins that are directly, or indirectly, associated with cell death or cell cellular longevity, including annexin A4, galectin-1, PDIA6, GDIA2, and syntenin-1 (Fig 3).

Annexin A4 shares 97% homology with annexin A5 thus similar mechanisms in the prevention of apoptotic cell phagocytosis by phosphatidylserine blockade have been proposed [30]. Annexin A4 activity is calcium-dependent and with increased levels of calcium upregulates the transcription of NFκB [31], which regulates apoptotic gene expressions.

Studies based on monocyte physiology in response to galectin-1 have shown opposing results as to whether galectin-1 induces apoptosis [32] or not [33]. Murine models have observed moderate increases of galectin-1 in atherosclerotic plaque suggesting a protective role [34]. The reduced expression seen in oxysterol treated THP-1 macrophages may be aiding apoptotic signalling in atherosclerosis.

In the present study, treatment with oxysterols caused a down-regulation of PDIA6 in THP-1 macrophages. PDI modification by oxLDL or by reactive carbonyls inhibits its enzymatic activity and potentiates both endoplasmic reticulum stress and apoptosis by oxLDL [35]. Inactivation or knock-down of PDI, including PDIA6, have also been associated with endoplasmic reticulum stress potentiating apoptosis [36].

Other proteins that less abundant in this functional group include GDIA2 and syntenin-1, which play roles in apoptosis and autophagy, respectively. Caspase-3 dependent cleavage of GDIA2 is seen to potentiate the progression of apoptosis [37]. Whereas the phosphorylation of syntenin-1, downstream of Ulk1, can potentiate autophagy [38].

### Proteins related to lipid-metabolism

Several proteins associated with lipid binding and metabolism were identified. This group includes proteins that were more abundant: macrophage scavenger receptor types I and II (MSR1), ATP5B, and ZNF202; and less abundant: cyclophilin A.

PMA differentiated THP-1 macrophages are known to have high basal levels of MSR1 expression [39]. MSR1, a class A type scavenger receptor, facilitates the ingestion of oxLDL, subsequent accumulation, and formation of foam cells, as well as inflammation and apoptosis [40]. Overexpression of MSR1 can also promote VSMCs apoptosis [41]. Caveolae-dependent endocytosis is required for class A macrophage scavenger receptor-mediated apoptosis in macrophages [42]. Recently, it has been shown that MSR1 expression can be pharmacologically modulated in macrophages [43]. In corroboration our results in both 2-DE and western blot analysis show the overexpression of MSR1 in THP-1 macrophages upon oxysterol treatment. This may contribute towards the increased lipid uptake, retention, and cell death previously observed in this cell model [23].

ATP5B has also been documented to play a role in lipid metabolism as well as cell death, as previously described above. In endothelial cells ATP5B’s function has been described in relation to cholesterol trafficking via the interaction with caveolae [44]. However, further clarification as to whether ATP5B colocalises with caveolin-1 in macrophages and the functional implications are to be examined.

Recent studies have shown the implications of ZNF202, a transcriptional repressor, in lipid metabolism and efflux with specific relation to HDL [45, 46]. ZNF202 represses genes encoding proteins required for HDL homeostasis including apo gene clusters; apoA-I/C-III/A-IV/A-V and apoE/C-I/C-IV/C-II [46], and lipid efflux proteins; ABCA1 and ABCG1 [45]. In macrophages ZNF202 may repress the expression of apolipoprotein E and lipid efflux proteins, altering the cell capacity for reverse cholesterol transport [46]. The upregulation of ZNF202 in oxysterol loaded THP-1 macrophages may provide a cellular model to study for cholesterol transport and lipid metabolism in the cells.

Cyclophilin A as an inflammatory mediator and is described as proatherogenic [47, 48]. Regarding lipid metabolism increased cyclophilin A and LDL uptake has been attributed towards cyclophilin A regulation of scavenger receptors in mice [49]. However, the 2-DE results in the present study indicate that the exposure of oxysterols to macrophages lead to a significant decrease in cyclophilin A but an increase in MSR1 expression. The differential expressional of the two proteins in the cell model may be explained by the function of cyclophilin A in both antiapoptotic and proapoptotic signalling [47, 48].

### Inflammatory related proteins

Three inflammatory related proteins were identified with significant differential expression upon oxysterol treatment of THP1 macrophages (Fig 3). Those which were significantly less abundant on treatment included; glyoxalase 1 and adenylyl cyclase-associated protein 1 (CAP1), which have both recently been studied in regards to atherosclerosis and monocyte function, respectively. Glyoxalase 1 functions to inhibit oxidative stress and advanced glycation [50], thereby reducing inflammation. Although levels have been reported to be reduced in atherosclerotic plaques [51], overexpression of CAP1 in monocytes produces an increase in NFκB transcription related pro-inflammatory cytokines [52]. The final inflammatory protein in this group, tryptophan–tRNA ligase, was found more abundant upon oxysterol loading. This is a genetic marker of monocyte to macrophage maturation, and also inhibits shear-stress activated responses in endothelial cells [53].

Discrepancies in the expressions of CYPA and HYOU1 were evident in the present study when comparing the two mass spectrometry methods, 2-DE and MS/MS. This can be attributed to isoform specific differential expression, in the 2-DE results two distinct CYPA protein spots are found significantly reduced in expression, and thereby selected for identification. The MS/MS quantification shows a slight increase in CYPA expression, though this represents total CYPA expression as specific isoforms are not distinguishable and quantified cumulatively. Isoform dependent alterations in HYOU1 may also be present in the 2-DE pattern, whereby only one isoform was significantly altered and selected for the analysis. Further studies to differentiate proteins isoforms may elucidate the specificity of the significant alterations in these proteins.

It should also be noted that a cell line was utilised in this study, PMA-differentiated THP-1 monocytes, which inherently reduces the degree of macrophage heterogeneity compared to tissue macrophages found within human atheroma. A study comparing the phenotypes of primary monocyte-derived macrophages and PMA-differentiated THP-1 macrophages has highlighted differences in morphology and surface marker expressions upon differentiation [54]. However, in a culture protocol similar to of the present study, PMA-treatment followed by a rest period resulted in differentiated macrophages with similar morphology and surface marker expression to primary monocyte-derived macrophages [54].

## Conclusions

Exposure of oxysterols, in an atheroma-relevant mixture, to THP-1 macrophages has resulted in significant alterations to the macrophage proteome. By 2-DE and mass spectrometry techniques differential expression of proteins in the cell model have been associated with *i*) signalling imbalance in cell death and cellular longevity; *ii*) lipid uptake and metabolism in foam cells; and *iii*) inflammatory marker proteins. The presented findings highlight a new proteomic platform for further studies into the functional roles of oxysterols and macrophages in atherosclerosis, and present a cell model for future studies to modulate the macrophage proteome by potential anti-atherosclerotic agents.

## Supporting information

S1 Table Identification of proteins from oxysterol treated THP-1 macrophages by means of 2-DE and MALDI-TOF MS. Listed proteins display significant differential expression upon 2mix treatment, identified by 2-DE and MALDI-TOF MS*, followed by database matching to the human UniProt database. Spot number refers to those displayed in Fig 2.
(PDF)

S2 Table Statistical comparisons between different control treatments on THP-1 macrophages. Data was obtained from 2-DE quantification of relative spot abundances. Data presented is limited to those proteins identified that show significant alterations between untreated samples and 7-ketocholesterol (28 μM), cholesterol (28 μM) or ethanol (2.8 μL/mL). Results do not show any significant alterations in protein abundances that were not significantly exacerbated further, in the same direction, by treatment with 2mix.
(PDF)
