# Supplementary material for: Exposure to atheroma-relevant 7-oxysterols causes proteomic alterations in cell death, cellular longevity, and lipid metabolism in THP-1 macrophages
Source: PLoS One. 2017 Mar 28;12(3):e0174475. doi: 10.1371/journal.pone.0174475 (PMC5370125; doi:10.1371/journal.pone.0174475)
Supplement: S1 Table — Listed proteins display significant differential expression upon 2mix treatment, identified by 2-DE and MALDI-TOF MS*, followed by database matching to the human UniProt database. Spot number refers to those displayed in Fig 2. (PDF) [file pone.0174475.s001.pdf]

S1 Table. Identification of proteins from oxysterol treated THP-1 macrophages by means of 2-DE and MALDI-TOF MS. Listed proteins display significant differential expression upon 2mix treatment, identified by 2-DE and MALDI-TOF MS*, followed by database matching to the human UniProt database. Spot number refers to those displayed in Figure 2.

Spot N#	Protein name	UniProt accession N#	MOWSE score	pI[a]	Mw (kDa) [a]	Seq. cov. (%)	Peptides matched
1	Hypoxia up-regulated protein 1	Q9Y4L1	19393	5.1 (5.2)	130 (111)	18.2	14
2	Zinc finger protein 202	O95125	220	5.2 (5.6)	62 (75)	8.0	5
3	ATP synthase subunit β	P06576	46707	4.9 (5.2)	48 (56)	21.6	8
4	Protein disulphide-isomerase A6	B7Z254	3794	5.0 (5.0)	44 (48)	20.4	7
5	Macrophage scavenger receptors types I and II	P21757	2176	5.1 (5.6)	43 (49)	24.8	9
6	Geranylgeranyl transferase type 2 subunit β	P53611	915	4.7 (4.9)	35 (37)	16.3	4
7	Rho GDP-dissociation inhibitor 2	P52566	105	5.1 (5.0)	25 (23)	18.4	4
8	Glyoxalase 1	Q04760	581	4.9 (5.1)	23 (21)	31.0	5
9	Phosducin-like protein	Q13371	97.4	4.7 (4.6)	21 (34)	25.2	5
10	Galectin-1	P09382	22077	5.0 (5.3)	12 (15)	54.1	8
11	Alpha-glucosidase II subunit α	Q14697	78004	5.5 (5.7)	100 (106)	14.5	14
12	Annexin A4	P09525	1573	5.6 (5.8)	34 (36)	20.1	6
13	Tryptophan – tRNA ligase	P23381	1259	5.8 (5.8)	49 (53)	14.6	7
14	Nuclear factor of activated T cells, cytoplasmic 1	O95644	385	6.8 (6.5)	82 (101)	7.2	6
15	Tyrosine-protein phosphatase non-receptor type 11	Q06124	838	6.2 (6.8)	69 (68)	15.2	6
16	Histone deacetylase 2	Q92769	332	6.0 (5.6)	58 (55)	16.0	5
17	Adenylyl cyclase-associated protein 1	Q01518	577453	7.0 (8.2)	54 (52)	25.1	14
18	Syntenin-1	O00560	4859	6.6 (7.0)	30 (32)	20.5	5
19	Cyclophilin A	P62937	1335	7.4 (7.6)	14 (18)	36.4	8
20	Cyclophilin A	P62937	1118	7.6 (7.6)	14 (18)	40.0	7

[a] values in parentheses are the theoretical values for each protein.

* Identification was performed using spectral mass peak lists submitted to the MS-Fit search engine (https://prospector.ucsf.edu) and searched within the Swiss-Prot and UniProt databases. Database search parameters were set as; *Homo Sapiens,* mass tolerance < 75ppm, maximum one missed cleavage, constant modification of carbomidomethyl on cysteine and variable modification of oxidation on methionine.
