# Supplementary material for: Exposure to atheroma-relevant 7-oxysterols causes proteomic alterations in cell death, cellular longevity, and lipid metabolism in THP-1 macrophages
Source: PLoS One. 2017 Mar 28;12(3):e0174475. doi: 10.1371/journal.pone.0174475 (PMC5370125; doi:10.1371/journal.pone.0174475)
Supplement: S2 Table — Data was obtained from 2-DE quantification of relative spot abundances. Data presented is limited to those proteins identified that show significant alterations between untreated samples and 7-ketocholesterol (28 μM), cholesterol (28 μM) or ethanol (2.8 μL/mL). Results do not show any significant alterations in protein abundances that were not significantly exacerbated further, in the same direction, by treatment with 2mix. (PDF) [file pone.0174475.s002.pdf]

S2 Table. Statistical comparisons between different control treatments on THP-1 macrophages. Data was obtained from 2-DE quantification of relative spot abundances. Data presented is limited to those proteins identified that show significant alterations between untreated samples and 7-ketocholesterol (28 µM), cholesterol (28 µM) or ethanol (2.8 µL/mL). Results do not show any significant alterations in protein abundances that were not significantly exacerbated further, in the same direction, by treatment with 2mix.

**Protein name**	**UniProt accession n#**	**Gene name**	**THP-1 macrophage treatment comparisons[a]**							
			**Con/7keto**	**Con/Chol**	**Con/EtOH**	**7keto/2mix**	**Chol/2mix**	**EtOH/2mix**	**Con/2mix**	**Regulation**
Adenylyl cyclase-associated protein 1	Q01518	CAP1	*ns*	*ns*	0.05	0.034	0.034	0.034	**0.05**	Down
Annexin A4	P09525	ANXA4	*ns*	*ns*	0.05	0.034	0.034	0.034	**0.027**	Down
Histone deacetylase 2	Q92769	HDAC2	*ns*	*ns*	0.05	0.034	0.034	0.034	**0.05**	Up
Hypoxia up-regulated protein 1	Q9Y4L1	HYOU1	0.05	*ns*	0.05	0.034	0.034	0.034	**0.05**	Up
Macrophage scavenger receptor types I and II	P21757	MSR1	*ns*	*ns*	0.05	0.034	0.034	0.034	**0.014**	Up
Tryptophan - tRNA ligase	P23381	WARS	*ns*	*ns*	0.05	0.034	0.034	0.034	**0.014**	Up
Tyrosine-protein phosphatase non-receptor type 11	Q06124	PTPN11	*ns*	*ns*	0.05	0.034	0.034	0.034	**0.027**	Up
Syntenin-1	O00560	SYCL	*ns*	*ns*	0.05	0.034	0.034	0.034	**0.014**	Down

[a] statistical comparison between treatment groups by non-parametric Mann-Whitney *U* test, significance $p \leq 0.05$

ns - non-significant; Con - control untreated; 7keto - 7-ketocholesterol; Chol - cholesterol; EtOH - ethanol; 2mix - mixture of 7β-hydroxycholesterol and 7-ketocholesterol
